# Twenty-four-hour intraocular pressure profiles and gonioscopic findings after gonioscopy-assisted transluminal trabeculotomy in primary open-angle glaucoma: a retrospective case series

**DOI:** 10.3389/fmed.2026.1828554

**Published:** 2026-06-29

**Authors:** Xiao Tang, Zhengfang Wu, Suju Liu, Ping Yu, Liuzhi Zeng

**Affiliations:** 1 Department of Ophthalmology, Affiliated Hospital of Integrated Traditional Chinese and Western Medicine, Chengdu University of Traditional Chinese Medicine (Chengdu First People’s Hospital), Chengdu, Sichuan, China; 2 Chengdu University of Traditional Chinese Medicine, Chengdu, Sichuan, China

**Keywords:** 24-h intraocular pressure, 24-h intraocular pressure fluctuation, gonioscopy, gonioscopy-assisted transluminal trabeculotomy, minimally invasive glaucoma surgery, peak intraocular pressure, primary open-angle glaucoma

## Abstract

**Background:**

To investigate changes in 24-h intraocular pressure (IOP) profiles and gonioscopic findings before and after gonioscopy-assisted transluminal trabeculotomy (GATT) in patients with primary open-angle glaucoma (POAG).

**Methods:**

This retrospective case series included 15 patients (25 eyes) with POAG who underwent GATT at the Affiliated Hospital of Integrated Traditional Chinese and Western Medicine, Chengdu University of Traditional Chinese Medicine, between January 2019 and January 2022 and completed pre- and postoperative 24-h IOP monitoring. Postoperative monitoring was performed at 3–6 months after surgery, after IOP-lowering medications had been discontinued for at least 7 days. Patients were followed for 2 years. Outcomes included pre- and postoperative 24-h IOP parameters, office IOP and medication burden at baseline and 2 years, and gonioscopic findings.

**Results:**

The 24-h IOP peak decreased significantly from 26.16 ± 1.44 mmHg preoperatively to 21.16 ± 0.60 mmHg postoperatively (mean ± SD; *P* = 0.001). The trough IOP decreased from 17.52 ± 0.66 mmHg to 16.25 ± 0.42 mmHg (mean ± SD; *P* = 0.098). IOP fluctuation decreased significantly from 8.68 ± 0.98 mmHg to 4.91 ± 0.34 mmHg (mean ± SD; *P* < 0.001). Mean office IOP decreased from 21.58 ± 1.03 mmHg at baseline to 18.45 ± 0.51 mmHg at 2 years (mean ± SD; *P* = 0.005), while the median number of medications decreased from 3 (2, 4) to 0 (0, 1). Gonioscopy showed no significant change in Spaeth angle grading across quadrants. Peripheral anterior synechiae were significantly more frequent postoperatively in the nasal and temporal quadrants, while trabecular meshwork pigmentation grade changed significantly in the nasal, temporal, and superior quadrants.

**Conclusion:**

In this small retrospective case series of POAG eyes, GATT was associated with reductions in 24-h IOP peak and fluctuation, sustained office IOP lowering, and marked medication reduction. Gonioscopy showed stable overall angle configuration, more frequent postoperative PAS in the nasal and temporal quadrants, and postoperative changes in trabecular meshwork pigmentation grade in some quadrants. These findings should be interpreted cautiously and confirmed in larger prospective studies.

## Introduction

Primary open-angle glaucoma (POAG) is a chronic blinding disease characterized by progressive damage to retinal ganglion cells and the retinal nerve fiber layer. Substantial evidence indicates that elevated intraocular pressure (IOP) and its 24-h fluctuation are important risk factors for glaucoma progression (1–3), whereas increased resistance within the trabecular meshwork–Schlemm’s canal (TM–SC) outflow pathway is a key pathological basis for IOP elevation in POAG (4, 5). Accordingly, restoring the physiological aqueous outflow pathway has long been a major therapeutic goal in glaucoma management.

A single office-based IOP measurement cannot fully reflect an individual’s true IOP burden. Compared with office time-point measurements, 24-h IOP monitoring better captures circadian fluctuation and provides a more comprehensive assessment of IOP exposure (1, 2, 6). In particular, the 24-h IOP peak and fluctuation amplitude have been closely associated with structural optic nerve damage and visual field progression (2, 6). Thus, 24-h IOP monitoring has become an important tool for evaluating IOP burden and treatment efficacy. In addition, body position and circadian rhythm may influence IOP levels and fluctuation patterns (1, 2). To reduce systematic error related to measurement-condition variability in this retrospective study, improve reproducibility, and better reflect routine clinical conditions, all 24-h IOP assessments were performed in the sitting position.

Minimally invasive glaucoma surgery (MIGS) has developed rapidly in recent years, but its effects on the 24-h IOP profile remain insufficiently studied. Gonioscopy-assisted transluminal trabeculotomy (GATT) is an ab interno, Schlemm’s canal–based MIGS procedure that lowers IOP by performing a circumferential 360° trabeculotomy to reconstruct the conventional aqueous outflow pathway (7). Previous studies have shown that GATT has favorable safety and sustained IOP-lowering efficacy in open-angle glaucomas such as POAG and juvenile-onset open-angle glaucoma, while also significantly reducing postoperative medication use (8–10). However, most existing studies have primarily focused on single postoperative office IOP measurements or mean IOP as the main outcome measures. Systematic evaluation of the 24-h IOP profile after GATT, particularly changes in peak IOP and fluctuation amplitude, remains limited. In addition, gonioscopic evidence regarding postoperative angle structural changes remains scarce.

Therefore, this study aimed to evaluate changes in the 24-h IOP profile before and after GATT in patients with POAG and to assess postoperative angle-related findings, including peripheral anterior synechiae (PAS), by gonioscopy. By integrating IOP dynamics with angle structural assessment, we sought to further characterize the effects of GATT on the conventional aqueous outflow pathway and provide additional evidence for its clinical application.

## Materials and methods

### Study design and participants

This was a single-center retrospective case series. Consecutive patients with primary open-angle glaucoma (POAG) who underwent gonioscopy-assisted transluminal trabeculotomy (GATT) at the Department of Ophthalmology, Affiliated Hospital of Integrated Traditional Chinese and Western Medicine, Chengdu University of Traditional Chinese Medicine, between January 2019 and January 2022 were included if they completed both preoperative and postoperative 24-h intraocular pressure (IOP) monitoring. A total of 15 patients (25 eyes) were enrolled. Both eyes from eligible patients were allowed to enter the study.

### Inclusion and exclusion criteria

The inclusion criteria were as follows: (1) age 18–85 years; (2) a clinical diagnosis of POAG and treatment with GATT; (3) inadequate IOP control despite standard antiglaucoma medical therapy before surgery; and (4) the ability to complete both preoperative and postoperative 24-h IOP monitoring. POAG was diagnosed based on the following criteria: an open angle on gonioscopy; IOP > 21 mmHg and ≤45 mmHg measured by Goldmann applanation tonometry, regardless of whether antiglaucoma medications were being used; glaucomatous structural optic nerve damage, such as neuroretinal rim thinning or notching, increased vertical cup-to-disk ratio, and/or inter-eye asymmetry; and characteristic glaucomatous visual field defects confirmed by reliable Humphrey visual field testing (SITA 24-2 or 30-2).

The exclusion criteria were as follows: (1) any history of prior intraocular surgery or ocular trauma; (2) receipt of any additional intraocular IOP-lowering surgical intervention before postoperative 24-h IOP monitoring; (3) other types of glaucoma, such as primary angle-closure glaucoma or secondary glaucoma, including pigmentary, steroid-induced, angle-recession, neovascular, inflammatory, or pseudoexfoliative glaucoma; (4) abnormal angle anatomy or anatomical conditions unsuitable for GATT; (5) severe ocular comorbidities affecting the acquisition of key parameters or the reliability of visual field testing; (6) severe systemic disease, pregnancy, or lactation; (7) severe psychiatric or psychological disorders affecting examination cooperation; and (8) incomplete follow-up data.

### Perioperative examinations and surgical procedure

All patients underwent routine preoperative slit-lamp examination, gonioscopy, fundus photography, visual field testing, OCT/OCTA, and 24-h IOP monitoring. All surgeries were performed by the same experienced chief surgeon. Patients were placed in the supine position and received topical anesthesia with proparacaine. Auxiliary clear corneal limbal incisions were created inferotemporally and superonasally with a 15° blade, and a main incision was made at the superotemporal clear corneal limbus using a 1.8 mm keratome. After miosis was induced, viscoelastic was injected into the anterior chamber. Under direct gonioscopic visualization, the trabecular meshwork was identified, and a Zeng’s trabeculotome was used to incise the trabecular meshwork and the inner wall of Schlemm’s canal by approximately 2 mm at around the 7:30 position. An iTrack microcatheter was then introduced into the anterior chamber through the auxiliary incision. Its tip was grasped with intraocular forceps and inserted into one end of the trabeculotomy opening into Schlemm’s canal, after which it was advanced circumferentially in a counterclockwise direction along the canal lumen. Viscoelastic was injected twice for approximately each clock hour of advancement. After 360° catheterization, the distal tip of the microcatheter was externalized through the opposite end of the trabeculotomy opening into the anterior chamber, and the other end of the catheter was pulled to achieve 360° circumferential trabeculotomy of the trabecular meshwork and inner wall of Schlemm’s canal. Complete 360° trabeculotomy was achieved in all included eyes. At the end of the procedure, residual viscoelastic and refluxed blood were irrigated from the anterior chamber, the corneal incisions were sealed in a watertight manner, and tobramycin–dexamethasone ointment was applied. Postoperatively, patients routinely received tobramycin–dexamethasone eye drops, or prednisolone acetate eye drops combined with antibiotic eye drops, diclofenac sodium eye drops, and sodium hyaluronate eye drops, for up to 2 weeks. Pilocarpine nitrate eye drops (2%) were started for miosis after active anterior chamber bleeding had resolved and were administered three times daily for 3 months. When further anti-inflammatory treatment was required after the initial steroid course, non-steroidal anti-inflammatory drugs were used with close monitoring of postoperative inflammation and IOP.

### Twenty-four-hour IOP monitoring protocol and definitions of IOP parameters

All 24-h IOP measurements were performed by the same experienced examiner. All patients were admitted to the inpatient ward for 24-h IOP monitoring. Postoperative 24-h IOP monitoring was scheduled at 3–6 months after surgery, as this period was considered more suitable for assessing relatively stable postoperative IOP dynamics after the early postoperative inflammatory phase and short-term treatment adjustments had largely subsided. Preoperative 24-h IOP monitoring was performed under each patient’s existing IOP-lowering medication regimen, representing the medicated preoperative baseline in routine clinical practice. To minimize the influence of topical antiglaucoma medications on postoperative 24-h IOP measurements, all IOP-lowering medications were discontinued for at least 7 days before postoperative monitoring. With reference to previous studies and reviews on 24-h IOP monitoring (1, 2), all preoperative and postoperative measurements were performed in the sitting position to ensure consistency of measurement conditions and improve reproducibility. IOP was measured using Goldmann applanation tonometry. Measurements were obtained every 2 h beginning at 8:00 a.m., for a total of 12 time points (08:00, 10:00, 12:00, 14:00, 16:00, 18:00, 20:00, 22:00, 24:00, 02:00, 04:00, and 06:00). At each time point, three consecutive measurements were obtained, and the mean value was used for analysis. After the 22:00 measurement, the lights were turned off and patients rested in the ward. For nighttime measurements, patients were awakened and IOP was measured immediately in the sitting position. The main 24-h IOP parameters were defined as follows: (1) peak IOP, the highest IOP value among the 12 time points (2); (2) trough IOP, the lowest IOP value among the 12 time points (2); and (3) 24-h IOP fluctuation, defined as peak IOP minus trough IOP.

### Gonioscopic examination

Preoperative and postoperative gonioscopy was performed by the same experienced examiner. Basic angle structure was recorded using the Spaeth grading system, including angle width, iris root insertion, peripheral iris configuration, and trabecular meshwork pigmentation (11–13). In addition, the presence and distribution of peripheral anterior synechiae (PAS) in each quadrant were recorded before and after GATT.

### Outcome measures

The primary outcome was the change in 24-h IOP parameters, including peak IOP, trough IOP, and 24-h IOP fluctuation, between baseline and 3–6 months postoperatively. Secondary outcomes included changes in office IOP and the number of IOP-lowering medications from baseline to 2 years postoperatively. Medications were counted according to the number of active ingredients, with each active ingredient counted as one medication; combination preparations were counted according to the number of constituent active ingredients. Additional secondary outcomes included changes in gonioscopic findings before and after surgery, including Spaeth grading, PAS, and trabecular meshwork pigmentation.

### Statistical analysis

Statistical analyses were performed using SPSS version 27.0. Continuous variables were presented as mean ± standard deviation or median (interquartile range), as appropriate according to data distribution. For paired preoperative and postoperative comparisons, normally distributed variables were analyzed using the paired-samples *t*-test, whereas non-normally distributed variables were analyzed using the Wilcoxon signed-rank test. Gonioscopy-related categorical data were presented as counts and percentages. Paired categorical gonioscopic outcomes were analyzed using McNemar’s exact test for binary variables and the Wilcoxon signed-rank test for ordinal variables. To evaluate the potential influence of inter-eye correlation, a one-eye-per-patient sensitivity analysis was performed. For patients with bilateral eligibility, only one eye was included in this sensitivity analysis. All statistical tests were two-sided, and a *P*-value < 0.05 was considered statistically significant.

### Ethics statement

This study was approved by the Ethics Committee of the Affiliated Hospital of Integrated Traditional Chinese and Western Medicine, Chengdu University of Traditional Chinese Medicine (Approval No. 2024YJS-019) and was conducted in accordance with the principles of the Declaration of Helsinki.

## Results

### Baseline characteristics

A total of 15 patients (25 eyes) with POAG were included, comprising 9 male patients (17 eyes) and 6 female patients (8 eyes), with a male-to-female ratio of approximately 2:1. The patients ranged in age from 23 to 74 years, with a mean age of 51.48 ± 17.29 years. According to disease stage, 3 eyes (12.0%) were classified as early stage, 8 eyes (32.0%) as moderate stage, and 14 eyes (56.0%) as advanced stage.

### Comparison of 24-h IOP parameters before and after GATT

All patients completed postoperative 24-h IOP monitoring at 3–6 months after surgery. On the day of preoperative 24-h IOP monitoring, patients were using a median of 3 (2, 4) IOP-lowering medications. Before postoperative monitoring, all IOP-lowering medications were discontinued for at least 7 days. Compared with preoperative values, the postoperative 24-h IOP peak decreased from 26.16 ± 1.44 mmHg to 21.16 ± 0.60 mmHg (mean ± SD), with a statistically significant difference (*P* = 0.001). The postoperative 24-h IOP trough decreased from 17.52 ± 0.66 mmHg to 16.25 ± 0.42 mmHg (mean ± SD), but this difference was not statistically significant (*P* = 0.098). The 24-h IOP fluctuation decreased from 8.68 ± 0.98 mmHg to 4.91 ± 0.34 mmHg (mean ± SD), with a statistically significant difference (*P* < 0.001) (Table 1).

**TABLE 1:** Comparison of 24-h IOP parameters before and after GATT (mmHg).

Group	24 h peak IOP	24 h trough IOP	24 h IOP fluctuation
Pre-op	26.16 ± 1.44 (23.18–29.14)	17.52 ± 0.66 (16.15–18.89)	8.68 ± 0.98 (6.66–10.70)
Post-op	21.16 ± 0.60 (19.92–22.40)	16.25 ± 0.42 (15.38–17.13)	4.91 ± 0.34 (4.22–5.60)
t	3.322	1.654	3.596
*p*	0.001	0.098	<0.001

Data are presented as mean ± standard deviation; values in parentheses indicate 95% confidence intervals. IOP, intraocular pressure; GATT, gonioscopy-assisted transluminal trabeculotomy.

**FIGURE 1:**
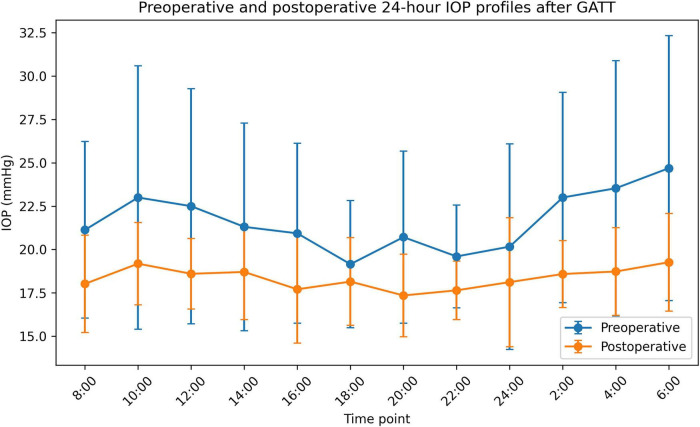
Preoperative and postoperative 24-h intraocular pressure profiles after GATT. The line graph shows mean IOP values at 12 time points before and after surgery. Error bars indicate standard deviation.

To provide a more complete overview of the 24-h IOP profile, the preoperative and postoperative IOP values at all 12 time points were plotted. The postoperative curve showed generally lower IOP values than the preoperative curve across most time points, which was consistent with the observed reductions in peak IOP and 24-h IOP fluctuation (Figure 1).

In the one-eye-per-patient sensitivity analysis, 15 eyes from 15 patients were included. The direction of changes in 24-h IOP parameters was generally consistent with the primary eye-level analysis. Peak IOP decreased from 26.47 ± 8.31 mmHg preoperatively to 21.25 ± 3.01 mmHg postoperatively (mean ± SD; *P* = 0.025), and IOP fluctuation decreased from 9.00 ± 5.50 mmHg to 5.16 ± 1.57 mmHg (mean ± SD; *P* = 0.020). Mean 24-h IOP also decreased from 21.87 ± 6.20 mmHg to 18.44 ± 2.51 mmHg (mean ± SD; *P* = 0.107), although this difference did not reach statistical significance in the restricted analysis, likely due to the reduced sample size.

### Comparison of office IOP and medication burden at baseline and 2 years

All 25 eyes completed the 24-month follow-up, and no eye required additional incisional glaucoma surgery during the 2-year follow-up period. The median number of IOP-lowering medications decreased from 3 (2, 4) preoperatively to 0 (0, 1) at 2 years postoperatively. Mean office IOP decreased from 21.58 ± 1.03 mmHg at baseline to 18.45 ± 0.51 mmHg at 2 years, with a statistically significant difference (*P* = 0.005).

### Comparison of gonioscopic findings before and after surgery

There were no statistically significant differences in Spaeth angle grading between the preoperative and postoperative examinations in any quadrant (all *P* > 0.05) (Table 2), indicating that the overall angle configuration remained stable after surgery.

**TABLE 2:** Comparison of Spaeth grading in each quadrant before and after GATT [*n* (%)].

Quadrant	Grade	Pre-op *n* (%)	Post-op *n* (%)	*P*
Nasal	A	1 (4.0)	0 (0)	0.180
	C	1 (4.0)	0 (0)	
	D	23 (92.0)	25 (100)	
Temporal	C	1 (4.0)	0 (0)	0.317
	D	24 (96.0)	25 (100)	
Superior	A	1 (4.0)	0 (0)	0.180
	C	1 (4.0)	0 (0)	
	D	23 (92.0)	25 (100)	
Inferior	B	0 (0)	2 (8.0)	0.234
	C	2 (8.0)	2 (8.0)	
	D	23 (92.0)	21 (84.0)	

Data are presented as *n* (%). *P*-values were calculated using the Wilcoxon signed-rank test for paired ordinal data.

The distribution of PAS changed after surgery. Paired analysis using McNemar’s exact test showed that PAS increased postoperatively in the nasal and temporal quadrants (*P* = 0.008 and *P* = 0.004, respectively), whereas no statistically significant changes were observed in the superior or inferior quadrants (*P* = 0.500 and *P* = 0.250, respectively) (Table 3).

**TABLE 3:** Comparison of the distribution of PAS before and after GATT [*n* (%)].

Quadrant	PAS	Pre-op *n* (%)	Post-op *n* (%)	*P*
Nasal	Absent	23 (92.0)	15 (60.0)	0.008
	Present	2 (8.0)	10 (40.0)	
Temporal	Absent	24 (96.0)	15 (60.0)	0.004
	Present	1 (4.0)	10 (40.0)	
Superior	Absent	22 (88.0)	20 (80.0)	0.500
	Present	3 (12.0)	5 (20.0)	
Inferior	Absent	24 (96.0)	22 (88.0)	0.250
	Present	1 (4.0)	3 (12.0)	

Data are presented as *n* (%). *P*-values were calculated using McNemar’s exact test for paired binary data.

Trabecular meshwork pigmentation grade was analyzed as an ordinal paired variable using the Wilcoxon signed-rank test. Significant postoperative changes in trabecular meshwork pigmentation grade were observed in the nasal, temporal, and superior quadrants (*P* = 0.033, *P* = 0.016, and *P* = 0.007, respectively), whereas the change in the inferior quadrant was not statistically significant (*P* = 0.514) (Table 4).

**TABLE 4:** Comparison of trabecular meshwork pigmentation grade in each quadrant before and after GATT [*n* (%)].

Quadrant	Grade	Pre-op *n* (%)	Post-op *n* (%)	*P*
Nasal	0	2 (8.0)	5 (20.0)	0.033
	1	5 (20.0)	9 (36.0)	
	2	17 (68.0)	10 (40.0)	
	3	1 (4.0)	1 (4.0)	
Temporal	0	1 (4.0)	5 (20.0)	0.016
	1	6 (24.0)	9 (36.0)	
	2	17 (68.0)	10 (40.0)	
	3	1 (4.0)	1 (4.0)	
Superior	0	1 (4.0)	5 (20.0)	0.007
	1	5 (20.0)	10 (40.0)	
	2	18 (72.0)	9 (36.0)	
	3	1 (4.0)	1 (4.0)	
Inferior	0	1 (4.0)	4 (16.0)	0.514
	1	5 (20.0)	7 (28.0)	
	2	18 (72.0)	9 (36.0)	
	3	1 (4.0)	4 (16.0)	
	4	0 (0)	1 (4.0)	

Data are presented as *n* (%). *P*-values were calculated using the Wilcoxon signed-rank test for paired ordinal data.

Because of the limited sample size, no formal subgroup statistical testing was performed to evaluate PAS-associated outcomes. On descriptive review, postoperative PAS in the nasal or temporal quadrants did not show a consistent apparent association with higher postoperative IOP, greater medication requirement, or incomplete trabeculotomy.

## Discussion

This retrospective study evaluated 24-h IOP characteristics and gonioscopic findings after GATT in patients with POAG. Our results showed that GATT was associated with reductions in the 24-h IOP peak and 24-h IOP fluctuation, while maintaining lower office IOP with a reduced medication burden. Gonioscopy demonstrated stable overall Spaeth angle grading after surgery, while postoperative PAS was more frequently observed in the nasal and temporal quadrants. In addition, trabecular meshwork pigmentation grade showed postoperative changes in the nasal, temporal, and superior quadrants. Taken together, these findings suggest that GATT may be associated with favorable improvements in postoperative IOP dynamics while producing characteristic postoperative gonioscopic changes. Given the retrospective design and small sample size, these findings should be interpreted as preliminary and hypothesis-generating.

This pathophysiological interpretation is in line with current understanding of POAG. Increased resistance within the TM–SC pathway, driven by fibrotic remodeling and increased tissue stiffness, is considered a major contributor to IOP elevation and fluctuation (4, 5). By creating a circumferential 360° trabeculotomy, GATT directly targets this key site of outflow resistance, allowing aqueous humor to bypass the diseased trabecular meshwork and enter Schlemm’s canal and the distal collector system (7). Previous studies have demonstrated sustained IOP reduction and medication sparing after GATT across several glaucoma subtypes, including POAG, juvenile-onset open-angle glaucoma, and steroid-induced glaucoma (8–10, 14). Our findings in an adult POAG cohort further support the potential real-world effectiveness of GATT.

Beyond static office IOP measurements, the 24-h IOP profile has increasingly been recognized as an important determinant of glaucoma progression. Previous studies have shown that higher mean 24-h IOP, higher peak IOP, and greater 24-h IOP fluctuation are associated with faster retinal nerve fiber layer thinning and worsening visual field loss (1, 2, 6). Body position and circadian rhythm also influence IOP levels and fluctuation patterns (1, 2), and a substantial proportion of IOP peaks occur outside routine clinic hours, suggesting that office measurements may underestimate the true IOP burden (1, 2). Therefore, 24-h IOP monitoring provides a more comprehensive assessment of postoperative IOP exposure and fluctuation and offers additional information for treatment evaluation and risk stratification (2, 6). To minimize measurement-related variability in this retrospective study, all preoperative and postoperative 24-h IOP assessments were performed in the sitting position. In our cohort, GATT significantly reduced both peak IOP and 24-h IOP fluctuation, whereas trough IOP showed only a mild, nonsignificant decrease. This pattern suggests that GATT may be particularly effective in blunting IOP peaks and stabilizing the overall IOP profile.

Notably, most patients in this study required multiple medications preoperatively to maintain IOP control, whereas by 2 years after surgery, the great majority required no medication or only minimal topical therapy while maintaining lower office IOP. This finding highlights the medication-sparing effect of GATT. Multiple long-term studies have reported a similar pattern of sustained IOP reduction accompanied by a marked reduction in medication use (8–10, 14). Grover et al. (7, 8) reported that GATT, as an ab interno 360° trabeculotomy, reduces outflow resistance at the level of the trabecular meshwork and inner wall of Schlemm’s canal, thereby lowering both IOP and medication burden and maintaining relatively stable pressure control through 24 months of follow-up.

An important consideration is that the preoperative and postoperative 24-h IOP assessments were not performed under identical medication conditions. Preoperative monitoring was performed under each patient’s existing IOP-lowering medication regimen, representing the medicated preoperative baseline in routine clinical practice, whereas postoperative monitoring was performed after discontinuation of IOP-lowering medications for at least 7 days. Therefore, the comparison should not be regarded as a strictly medication-matched assessment. This difference may have influenced the magnitude of IOP reduction and should be considered when interpreting the surgical effect. Nevertheless, the lower postoperative peak IOP and IOP fluctuation despite discontinuation of IOP-lowering medications before postoperative monitoring may support a potential surgical effect under real-world clinical conditions, although this interpretation should remain cautious.

Our gonioscopic assessment provides complementary structural information regarding postoperative angle status after GATT. In this study, overall Spaeth grading remained stable, indicating that the overall angle configuration remained open and did not show significant postoperative narrowing. PAS was more frequently observed postoperatively in the nasal and temporal quadrants. These changes may be related to localized postoperative angle adhesion or remodeling, and their overall extent appeared limited. On descriptive review, postoperative PAS did not show a consistent apparent association with higher postoperative IOP, greater medication requirement, or incomplete trabeculotomy in this small cohort. Previous studies have also suggested that limited PAS after GATT may not substantially compromise long-term IOP control (10, 14). Nevertheless, the clinical significance of postoperative PAS remains uncertain and should be further investigated in larger studies. In addition, paired analysis showed postoperative changes in trabecular meshwork pigmentation grade in the nasal, temporal, and superior quadrants. These changes may reflect postoperative redistribution of pigment, altered gonioscopic visibility after trabeculotomy, local changes in the trabecular meshwork region, or variability inherent to semi-quantitative gonioscopic grading (11–13). Because pigmentation grading was based on retrospective gonioscopic assessment and the sample size was small, this finding should be interpreted cautiously. Future prospective studies incorporating standardized gonioscopic documentation and quantitative anterior segment imaging are needed to clarify whether postoperative pigmentation changes are associated with outflow remodeling or long-term IOP control.

External evidence from anterior segment imaging and aqueous outflow studies further suggests that trabeculotomy-based angle surgery may induce structural and functional changes in the conventional outflow pathway. Yoshikawa et al. reported that anterior segment OCT after trabecular meshwork surgery demonstrated greater openness of outflow-related structures together with altered outflow patterns (15). Dada et al., using aqueous angiography, demonstrated recruitment of aqueous outflow channels after ab-interno goniectomy (16). When considered together with our 24-h IOP and gonioscopic findings, these observations are consistent with the interpretation that GATT may improve postoperative IOP control by reducing resistance in the conventional aqueous outflow pathway, although this mechanism cannot be directly proven by the present retrospective study.

The one-eye-per-patient sensitivity analysis further supported the robustness of the 24-h IOP findings, as the overall direction of changes was consistent with the primary eye-level analysis. Peak IOP and IOP fluctuation remained significantly reduced after surgery, whereas the reduction in mean 24-h IOP did not reach statistical significance after restricting the analysis to one eye per patient. This finding may reflect reduced statistical power due to the smaller sample size and highlights the need for larger prospective studies. Therefore, the present findings should be interpreted as preliminary and hypothesis-generating rather than definitive evidence of the effect of GATT on 24-h IOP dynamics.

Several limitations of this study should be acknowledged. First, this was a single-center retrospective case series with a relatively small sample size, which may limit the generalizability of the findings. Second, both eyes from some eligible patients were included in the primary eye-level analysis. Although a one-eye-per-patient sensitivity analysis was performed, the small sample size still limits statistical robustness. Third, the preoperative and postoperative 24-h IOP assessments were not medication-matched. Preoperative monitoring was performed under the existing medication regimen, whereas postoperative monitoring was performed after discontinuation of IOP-lowering medications for at least 7 days. This difference should be considered when interpreting the magnitude of postoperative IOP reduction. Fourth, although follow-up extended to 2 years, longer observation is still needed to determine the durability of the treatment effect. Fifth, visual acuity and visual field progression were not systematically analyzed in the present study. Although visual acuity and visual field examinations were performed as part of routine clinical follow-up, visual acuity was not predefined as a primary or secondary outcome, and some original visual field records could not be fully retrieved because of data storage limitations and loss of archived testing records. Therefore, formal longitudinal analyses of visual acuity change and visual field progression were not performed. Sixth, angle assessment relied mainly on gonioscopy, which is inherently subjective, and quantitative imaging modalities such as anterior segment OCT and aqueous angiography were not systematically incorporated. Future multicenter prospective studies with larger samples, medication-matched 24-h IOP monitoring, appropriate methods to account for within-patient correlation, and quantitative angle imaging are warranted to further clarify the effects of GATT on IOP dynamics and outflow-related changes.

In conclusion, in this small single-center retrospective case series of POAG eyes, GATT was associated with reductions in 24-h IOP peak and fluctuation, sustained office IOP lowering, and marked medication reduction. Gonioscopy showed stable overall angle configuration but more frequent postoperative PAS in the nasal and temporal quadrants, together with postoperative changes in trabecular meshwork pigmentation grade in some quadrants. Given the retrospective design, small sample size, inclusion of both eyes in the primary analysis, and differences in medication status during 24-h IOP monitoring, these findings should be interpreted as preliminary and hypothesis-generating. Larger prospective studies are needed to confirm the effects of GATT on 24-h IOP dynamics and postoperative angle remodeling.

## Data Availability

The de-identified datasets used and/or analyzed during the current study are available from the corresponding author on reasonable request.
